# Meta-analysis of honey bee neurogenomic response links Deformed wing virus type A to precocious behavioral maturation

**DOI:** 10.1038/s41598-020-59808-4

**Published:** 2020-02-20

**Authors:** Ian M. Traniello, Syed Abbas Bukhari, Jessica Kevill, Amy Cash Ahmed, Adam R. Hamilton, Nicholas L. Naeger, Declan C. Schroeder, Gene E. Robinson

**Affiliations:** 1 Neuroscience Program, University of Illinois at Urbana-Champaign, (UIUC), Urbana, IL USA; 2 Carl R. Woese Institute for Genomic Biology, UIUC, Urbana, USA; 3 Department of Animal Biology, UIUC, Urbana, USA; 4 Department of Veterinary Population Medicine, University of Minnesota, St. Paul, MN USA; 5 Department of Entomology, Washington State University, Pullman, WA USA; 6 School of Biological Sciences, University of Reading, Reading, UK; 7 Department of Entomology, UIUC, Urbana, USA

**Keywords:** Agroecology, Agroecology, Social behaviour, Social behaviour

## Abstract

Crop pollination by the western honey bee *Apis mellifera* is vital to agriculture but threatened by alarmingly high levels of colony mortality, especially in Europe and North America. Colony loss is due, in part, to the high viral loads of Deformed wing virus (DWV), transmitted by the ectoparasitic mite *Varroa destructor*, especially throughout the overwintering period of a honey bee colony. Covert DWV infection is commonplace and has been causally linked to precocious foraging, which itself has been linked to colony loss. Taking advantage of four brain transcriptome studies that unexpectedly revealed evidence of covert DWV-A infection, we set out to explore whether this effect is due to DWV-A mimicking naturally occurring changes in brain gene expression that are associated with behavioral maturation. Consistent with this hypothesis, we found that brain gene expression profiles of DWV-A infected bees resembled those of foragers, even in individuals that were much younger than typical foragers. In addition, brain transcriptional regulatory network analysis revealed a positive association between DWV-A infection and transcription factors previously associated with honey bee foraging behavior. Surprisingly, single-cell RNA-Sequencing implicated glia, not neurons, in this effect; there are relatively few glial cells in the insect brain and they are rarely associated with behavioral plasticity. Covert DWV-A infection also has been linked to impaired learning, which together with precocious foraging can lead to increased occurrence of infected bees from one colony mistakenly entering another colony, especially under crowded modern apiary conditions. These findings provide new insights into the mechanisms by which DWV-A affects honey bee health and colony survival.

## Introduction

Western honey bees (*Apis mellifera*) provide an essential pollination service for modern agriculture, with a value estimated at $15 billion per year in the United States^1,2^. Extensive reliance on pollination by honey bees has led to great concerns over the massive losses of managed colonies that have occurred since 2006^3–5^. These losses are widely thought to be caused by parasites, pathogens, pesticides and poor nutrition, and the main parasite is the ectoparasitic mite *Varroa destructor*^6–10^. *Varroa* mites are the primary vectors of Deformed wing virus (DWV)^11,12^, a single-stranded, positive-sense RNA virus (family Iflaviridae) that specifically impacts arthropods^13–16^.

DWV is globally distributed, driving an economic and ecological crisis in honey bee populations^17^. DWV was first discovered in symptomatic deformed winged honey bees from Japan in the early 1980s^18^, and the first full genome was sequenced in 2006^19^, hereafter referred to as type A or DWV-A^20^. Martin *et al*. showed through screening the Hawaiian honey bee population that diverse DWV variants persisted prior to the arrival of *Varroa*^21^; however, the establishment of *Varroa* selected for a single variant, DWV-A. DWV-A is currently the most prevalent variant in the North America^22^, though two other master variants have been identified: DWV-B, which is more commonly found in Europe and is also associated with colony loss^22,23^, and DWV-C, which is extremely rare in North America, though its effects are currently unknown^20,22,24^.

DWV localizes to major sensory and behavioral centers of the bee brain where it actively replicates^25^, and behavioral consequences of infection on associative learning and reward responsiveness have been documented^26^. In addition, studies have demonstrated that low doses of DWV increase mortality rates, cause workers to accelerate their behavioral maturation and initiate foraging at younger ages, and forage less than controls despite no physical symptoms, supporting previous correlative findings^27,28^. These behavioral studies imply a disruption of critical behavioral programs, suggesting that viral infection has multiple and strong effects on brain and behavior. Neurogenomic studies might be helpful to understand the mechanistic basis of these effects, but no study has yet examined DWV effects on brain gene expression in adult worker honey bees.

**Figure 1:**
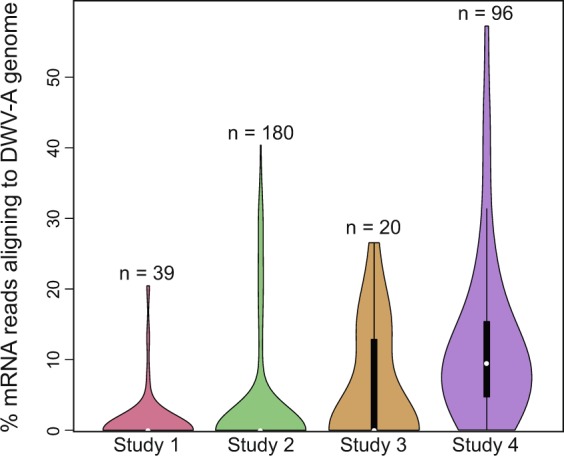
Percentage of mRNA sequencing reads found in honey bee whole-brain and mushroom bodies RNA extractions that aligned to the DWV-A viral genome (accession number AJ489744) instead of the honey bee genome (version HAv3.1). Studies are organized by average % mRNA reads aligning to DWV genome, from smallest to largest. Studies 1–3 performed RNA-Sequencing (RNA-Seq) from the mushroom bodies of adult honey bee brains while Study 4 utilized the whole honey bee brain for RNA-Seq (see Table 1 for study details). Width of the violin plot is proportional to frequency of samples that fall in a given region on the y-axis. Boxplots show median (white half-circle or circle at densest point of violin plot), quartiles (thick black bar) and 0.05 and 0.95 quantiles (thin black lines). Sample sizes, reported above each violin plot, refer to the number of individual bees analyzed. Raw read data can be found in Figshare repository (see below).

Honey bees can be infected naturally with high DWV titers without displaying obvious, physical symptoms^27,28^. To explore mechanistic implications, we took advantage of four brain transcriptome studies that unexpectedly revealed evidence of covert DWV-A infection, two published^29,30^ and two new RNA-Sequencing (RNA-Seq) datasets (Table 1). In these datasets over 99% of 335 individual brain samples contained at least one sequenced read aligned to the DWV-A viral genome, rather than the honey bee genome^19^, and more than one third of the samples contained over 1% of sequenced reads aligned to DWV-A (Fig. 1). All bees collected for these studies were negative for any visible symptoms of DWV^19,31^ and no overt signs of impairment were observed in specific behavioral paradigms. We performed gene expression and transcriptional regulatory network analyses to compare these four RNA-Seq datasets and explore how DWV-A affects brain gene expression, as a step towards implicating specific molecular pathways associated with the global decline in bee health. The existence of multiple transcriptome datasets afforded the opportunity to test for consistent, robust transcriptome signatures of viral infection across the four otherwise unrelated studies and to determine whether DWV-A targets pathways similar to other bee viruses^32^.

**Table 1:** Overview of the honey bee brain transcriptome studies included in this meta-analysis.

Study	Sample size/caste	Age	Brain region	Queen	GEO Accession	Year of sample collection	Publication	Focal behavior
1	39/workers	2–3 weeks	Mushroom bodies	Naturally mated	GSE130701	2012	McNeill *et al*. 2015	Reward responsiveness
2	180/workers	7 days	Mushroom bodies	Single-drone inseminated	GSE85878	2014	Shpigler *et al*. 2017	Aggression
3	20/drones	2–3 weeks	Mushroom bodies	Naturally mated	GSE130702	2012	Unpublished	Reward responsiveness
4	96/workers	6 days	Whole brain	Single-drone inseminated	GSE130700	2014	Unpublished	Caregiving/affiliation

We analyzed two published (Studies 1 and 2) and two new (Studies 3 and 4) brain transcriptome studies which were independently performed in apiaries in Urbana, IL from 2012 to 2014.

We tested the hypothesis that DWV-A affects some of the same molecular pathways that influence behavioral maturation, as covert DWV-A infection has been shown to cause bees to forage at a younger age than normal^33^. Worker honey bees undergo an age-related transition from in-hive nurses to out-of-hive foragers, and this process of behavioral maturation can be accelerated or delayed depending on the needs of the colony^34^. The effect of DWV-A infection on behavioral maturation is of particular importance because precocious behavioral maturation has independently been associated with diminished spatial memory and colony failure^33,35,36^, so disoriented foragers may be more likely to spread DWV to neighboring colonies. Therefore, laying the groundwork for a mechanistic link between viral infection and accelerated behavioral maturation could provide new insights into the dynamics of this host-pathogen relationship.

## Results

### Common patterns of brain gene expression in DWV-A infected bees

To examine the molecular consequences of DWV-A infection in the honey bee brain, we analyzed two published^29,30^ and two new brain RNA-Seq datasets containing transcriptome data from both adult worker (female) and drone (male) honey bees (Table 1). We used DWV-mapped transcripts as a linear predictor of brain gene expression (Fig. 1). Using a false discovery rate (FDR) < 0.05, we found the following numbers of positively and negatively DWV-A associated genes, hereafter referred to as “DWV-associated genes”: 77 positively and 0 negatively associated (Study 1^29^); 1072 positively and 695 negatively associated (Study 2^30^); 134 positively and 162 negatively associated (Study 3); and 137 positively and 232 negatively associated (Study 4; all gene lists in Supplementary Tables S1–S4). In Study 2^30^, the authors excluded from post-sequencing analysis 28 samples with DWV-A infection found to be “severe” (having more than 0.5% DWV-mapped transcripts); here, we have included them. In Study 4, several behavioral and molecular perturbations were conducted but did not result in any differential gene expression between groups, which we attribute to DWV-A infection. We have included all the samples from that study and deposited all relevant treatment information with the GEO submission (GSE130700).

**Figure 2:**
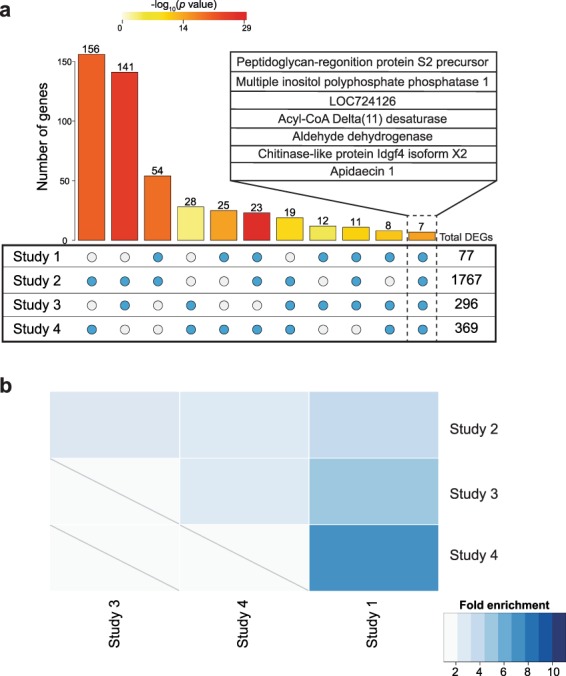
DWV-associated brain gene expression is highly similar between studies. (**a**) Hundreds of DWV-associated genes were shared between at least two independent studies, with a list of seven genes associated with all four studies (inset). (**b**) Results of hypergeometric tests show significant overlap of DWV-associated genes across various combinations of the four studies. A gene list universe common to all four studies was used to increase stringency of analysis. Studies are ordered by increasing fold enrichment score (see Methods). All pairwise comparisons were significant at FDR < 0.0005 (raw and corrected *p* values in Supplementary Table S5).

Calculating all possible intersections among the four datasets we found seven DWV-associated genes shared between all datasets (hypergeometric test, fold enrichment [FE] = 290.59, *p* < 0.01, Fig. 2a): peptidoglycan-recognition protein S2 precursor (*PGRP-S2*), multiple inositol polyphosphate phosphatase 1, uncharacterized LOC724126, acyl-CoA delta-11 desaturase, aldehyde dehydrogenase (*aldh*), chitinase-like protein Idgf4 isoform X2 (*Idgf4*), and apidaecin 1 (*apid1*). Each gene was positively associated with DWV-A infection except for uncharacterized LOC724126 in Study 3, which was negatively associated.

**Figure 3:**
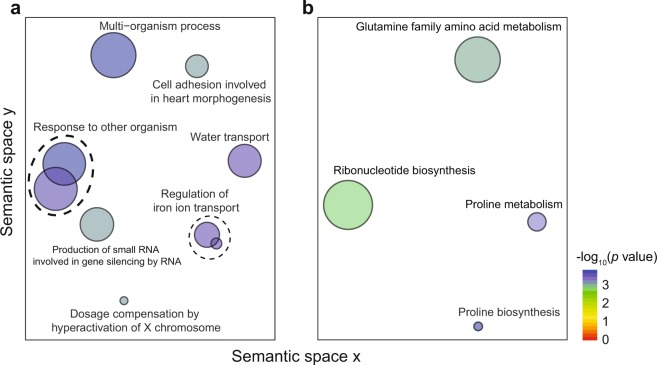
REVIGO plots show Gene Ontology (GO) terms enriched in (**a**) positively or (**b**) negatively associated DWV-associated gene lists. Plots are organized in semantic space in which more similar terms in the GO hierarchy are more closely positioned. Circle diameter is correlated with the inverse of the GO term’s specificity with smaller circles representing more specific terms.

We found a significant overlap for each pairwise comparison among the four DWV-associated gene lists (hypergeometric test, FE > 2.0, FDR < 0.01 for each pairwise comparison, Fig. 2b; Supplementary Table S5). Gene Ontology (GO) enrichment analysis performed on genes positively associated with DWV-A with GOrilla^37^ identified an over-representation of several GO terms associated with iron metabolism, response to virus, and other biological processes (Fig. 3a; Supplementary Table S6). We found fewer GO terms enriched in genes negatively associated with DWV-A, primarily involved with amino acid synthesis and metabolism (Fig. 3b; Supplementary Table S7). GO terms for individual datasets are in Supplementary Tables S8–S15.

### DWV-associated gene expression compared to previous studies of nurse-forager gene expression

To test the hypothesis that DWV-A influences the molecular pathways underlying behavioral maturation and accelerates the nurse-to-forager transition, we compared DWV-associated gene expression in each study to published transcriptomic comparisons of nurses and foragers. None of the four studies we analyzed included comparisons of nurses and foragers, although Study 4 examined caregiving (nursing) behavior among bees. This was examined in small, age-matched groups and not as a naturally occurring behavior in the field. Also, no comparisons were made to foragers or older bees of any behavioral state and, moreover, we could not detect any effect of caregiving on gene expression profile (see above for information regarding Study 4, in which no differentially expressed genes were detected relative to experimental conditions). Studies 2 and 4 contained adult workers that were considerably younger than typical foraging age, the onset of which is at least 2–3 weeks of age (see Table 1)^38,39^. To obtain focal bees for these studies, honeycomb frames of capped brood were stored in an incubator and newly eclosed adults were collected every 24 hours. This method has been regularly used to form groups of bees in which the exact age is known^40,41^. Therefore, we suggest that behavioral maturation-related gene expression in DWV-A infected samples was specifically due to viral infection and not conflated with experimental conditions.

**Figure 4:**
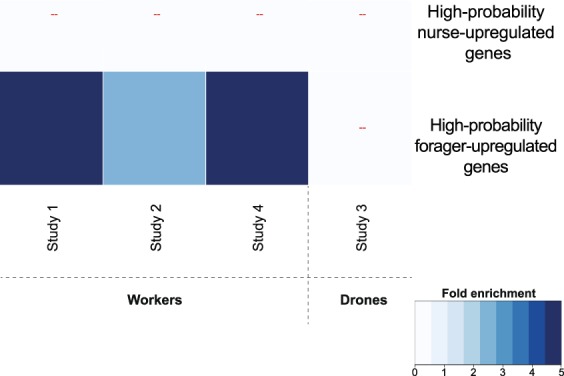
Neurogenomic state of DWV-A infected bees is more similar to foragers than nurses. Genes positively associated with DWV-A infection showed significant overlap with nurse- and forager-upregulated genes validated between two independent studies^42,43^, which we term “high-probability” genes. Pairwise comparisons were significant (FDR < 0.05) in Study 1, 2 and 4, which sampled (female) workers but not Study 3, which sampled (male) drones. FE, raw and corrected *p* values are reported in Supplementary Table S16. “–” denotes a nonsignificant overlap.

We used nurse- and forager-related transcriptome profiles previously validated across two published studies not analyzed here^42,43^. We only considered genes that were concordant across these two studies despite differences in technical and methodological details; given this stringency, we use these nurse- and forager-upregulated genes as designations of nursing and foraging behavioral states. In Studies 1, 2 and 4, which all contain worker (female) honey bees, we found that genes positively associated with DWV-A infection were significantly enriched in forager, but not nurse, transcriptome profiles (hypergeometric test, FE > 1.8, FDR < 0.05). Study 3, which contains drone (male) samples, did not show significant enrichment in nurse or forager gene lists (Fig. 4, Supplementary Table S16).

### Transcriptional regulatory network analysis

**Figure 5:**
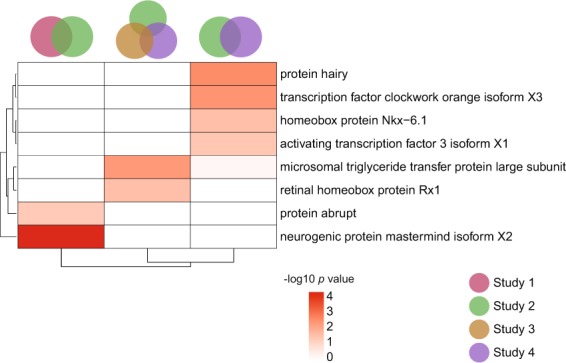
DWV-A infection influences the brain transcriptional regulatory architecture of brain gene expression. We used ASTRIX to infer a transcriptional regulatory network associated with DWV-A infection. We found eight transcription factors (TF) with target genes enriched in at least two datasets, implicating them as important regulators of the effects of DWV-A infection on honey bee brain gene expression.

To help identify transcription factors important in regulating the transcriptional response in the honey bee brain to DWV-A infection, we reconstructed a transcriptional regulatory network (TRN) using brain gene expression to infer regulatory interactions between transcription factors (TFs) and their targets. We only used orthologs of TFs and target genes that have been experimentally validated in *Drosophila melanogaster*^44^ and generated a TRN of 248 TFs and 5716 target genes (Supplementary Table S17). We found 19 TFs with predicted target genes significantly enriched in the DWV-associated gene lists, the majority of which (13/19) showed enrichment only in Study 2 (Supplementary Table S18). Eight TFs, including protein hairy (*hairy*), transcription factor clockwork orange isoform X3 (*cwo*), and activating transcription factor 3 isoform X1 (*atf3*) had their predicted target genes enriched in at least two DWV-associated gene lists (Fig. 5, Supplementary Table S19). Studies 2 and 4, which contained only young (age ≤ 7 days) worker bees collected in 2014, shared the most TFs.

### ABC and CBA assays for DWV-A subtype validation

**Figure 6:**
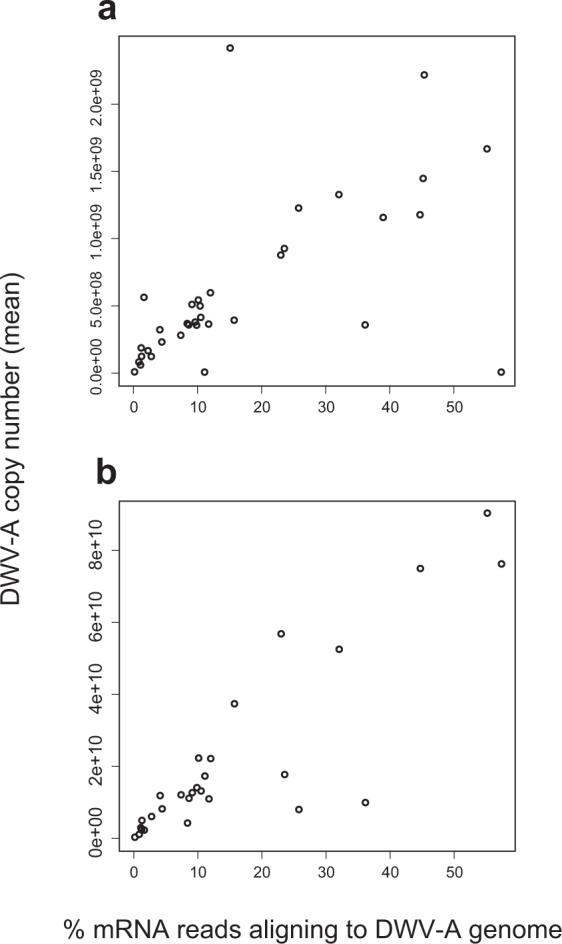
RT-PCR analyses uncover full-length DWV-A transcripts. (**a**) ABC assay, which targets the *RNA dependent RNA polymerase* (*RdRp*) region in DWV-A, provided mean copy numbers of master variant DWV-A which was significantly correlated with % reads aligned to DWV-A viral genome (r = 0.59, *p* < 0.001). (**b**) CBA assay, which targets the capsid region of DWV transcripts, showed a significant correlation for DWV-A (r = 0.87, *p* = 1.97e-09). Pearson correlation; n = 35 whole-brain samples from Study 4. We found no evidence for DWV type B or C.

To validate the read-mapping specificity to reliably discriminate between the master variants of DWV, we used two competitive reverse transcription polymerase chain reaction (RT-PCR) assays to target different portions of the virus genome (n = 35 samples from Study 4). Using the ABC assay^45^, which targets the *RNA dependent RNA polymerase* (*RdRp*) region in DWV, we found a significant correlation between %DWV and DWV type A (Pearson correlation; DWV-A, r = 0.59, *p* < 0.001, Fig. 6a). Next, we used the CBA assay [Highfield *et al*., *in preparation*] to target the DWV capsid region. Using this assay, we found a strong relationship between %DWV-A as identified through mapping and DWV-A through RT-PCR (r = 0.87, *p* = 1.97e-09, Fig. 6b). We did not find evidence of DWV type B or C in our honey bee samples.

### Single-cell RNA-Sequencing

**Figure 7:**
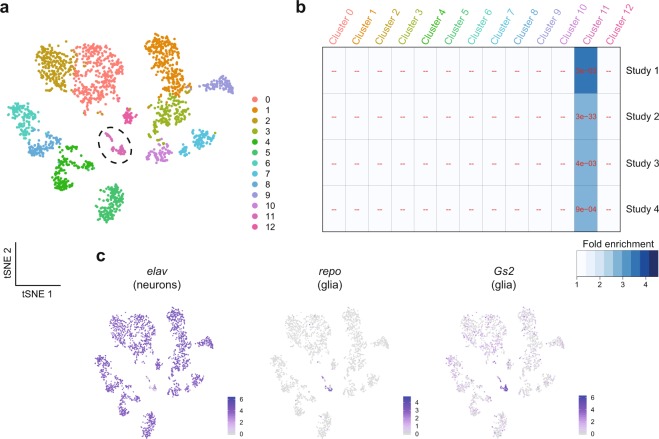
Single-cell RNA-Sequencing demonstrates significant enrichment of DWV-associated genes in a single predicted cluster of honey bee brain cells. (**a**) Two-dimensional visualization of single-cell RNA-Sequencing (scRNA-Seq) from one whole brain and one mushroom bodies from two healthy worker bees, independent of the bees used in the previous analyses. We used tSNE clustering to sort cells into 13 clusters based on gene expression profiles of 2,205 cells. Dashed circle outlines Cluster 11. (**b**) Hypergeometric overlap tests comparing positively associated DWV-associated genes and cluster-defining upregulated genes (see Methods) show that DWV-associated genes are only enriched in a single cluster, Cluster 11. All BH-corrected *p* values are < 0.005. “--” denotes a nonsignificant overlap. (**c**) The canonical neuronal marker *elav* is highly expressed in the vast majority clusters excluding Cluster 11, where glial markers *repo* and *Gs2* are specifically expressed. Color scales represent log-normalized gene expression per cell.

To identify specific cell populations in the brain impacted by DWV-A, we used the 10x Genomics (10x) Chromium platform to perform single-cell RNA-Sequencing (scRNA-Seq) on the whole brain (WB) and mushroom bodies (MB) of two individual bees unrelated to the studies analyzed here. We chose both WB and MB because both were sampled for the datasets analyzed in this meta-analysis (Table 1) and including a focal region (MB) might help identify rarer cells types we would otherwise be unable to identify. Each library generated over 360 million reads and we recovered transcriptome data from ~1,200 cells per sample with a median of 1,167 and 1,559 genes per cell, for the WB and MB, respectively (Fig. 7a). We merged both WB and MB datasets and identified 13 cell clusters based on similarities in gene expression profiles (Supplementary Table S20).

To test the hypothesis that the neurogenomic signature of DWV-A infection is localized to a specific brain region or cell subpopulation, we performed a series of hypergeometric tests to compare genes positively associated with DWV-A infection and genes upregulated per predicted cell cluster. We only found a significant result in Cluster 11, in which each DWV-associated gene list had a significant FE score (FDR < 0.01, Fig. 7b). The neuronal marker *elav* was largely absent from this cluster, whereas the insect glial markers reversed polarity (*repo*)^46^ and glutamine synthetase (*Gs2*)^47^ were significantly upregulated (Wilcoxon Rank Sum Test, *repo*: average log fold-change = 2.39, FDR = 1.61e-130; *Gs2*: average log fold-change = 3.22, FDR = 6.5e-31). Neither *repo* nor *Gs2* were upregulated in any other cluster (Fig. 7c).

## Discussion

We identified common transcriptional signatures of DWV-A infection in the honey bee brain based on brain transcriptome studies of asymptomatic individuals, thus presenting the first neurogenomic analysis of the conserved molecular impact of covert yet widespread DWV-A infection. Our results, which were due to infection exclusively with DWV type A, indicate strong effects on honey bee brain gene expression, with several molecular pathways and transcription factors implicated across multiple independent studies. In addition, our meta-analysis highlights strong similarities in brain gene expression and transcriptional regulatory architecture between infected bees and natural foragers, thus filling an important gap in our understanding of how DWV-A may impact colony demographics and survival. DWV-A appears to accelerate honey bee behavioral maturation and activate molecular pathways in the brain associated with the transition from nurse to forager.

Some of the genes identified here have been implicated in viral infection in other studies of honey bees. The antimicrobial peptide-encoding *apid1* and the pathogen recognition receptor *PGRP-S2*, which were positively associated with DWV-A infection in all four studies, have also been shown to be casually linked to dsRNA treatment or Sindbis virus infection in the thorax and abdomen of infected honey bees^48^ and therefore may be activated following generalized inflammation in multiple tissues. Moreover, a meta-analysis of 19 transcriptome datasets of experimentally infected worker honey bees, which did not include an analysis of DWV in the adult brain, found that PGRPs were either consistently differentially expressed or were predicted to regulate many genes across datasets^32^. *PGRP*-*S2* also was found to be upregulated in bees taken from colonies diagnosed with CCD^49^. GO enrichment analysis revealed that genes positively associated with DWV were enriched for gene silencing by RNA, which plays a role in insect antiviral defense^50^ and may therefore signify a protective mechanism against DWV infection.

Other genes identified here have been implicated in viral infection or inflammation across a broad range of conditions among phylogenetically diverse species, including humans. This is remarkable, given that insects lack lymphocytes, immunoglobulins and other fundamental units of the vertebrate immune system. For example, GO enrichment analysis revealed that genes positively associated with DWV-A were enriched for iron metabolism, and viral hijacking of iron metabolism has been associated with poor prognosis in HIV-1 and hepatitis C infection in humans, as iron accumulation provides an ideal environment for viral replication^51^. Another link to human disease was *aldh*, positively associated with DWV-A in all four studies, which was shown to offer a neuroprotective function in Parkinson’s disease-associated neurodegeneration of dopaminergic cells^52^.

We found *Idgf4*, a chitinase-related gene associated with chitin organization and cuticle development^53^, to be positively associated with DWV-A in all four studies. Chitinase and chitinase-related genes are an evolutionarily conserved family of hydrolases synthesized in both insects and mammals^54^. Though mammals do not synthesize chitin, these hydrolases have been implicated in the pathogenesis of inflammatory responses in several human disorders^55,56^, and expression of the mammalian chitinase-3-like protein 1 is increased in astrocytes following traumatic brain injury^57^. These results suggest that viral and host responses contain some deeply conserved components. Moreover, expression of *Idgf4* was shown to increase in the abdominal integuments with the onset of foraging in honey bees and another social bee species^58^, supporting our hypothesis that DWV-A infection affects molecular pathways associated with behavioral maturation. While *Idgf4* does not have chitinase activity, it likely promotes cell growth and development, the function of which is unclear in the post-mitotic adult brain^59^. *Idgf4* expression was positively correlated with sexual maturation in the neural ganglia of the female Pacific abalone *(Haliotis discus hannai*)^60^, implying that it has broad maturation-related functionality in different tissue types, including neural.

Our results suggest that DWV-A drives a forager-like neurogenomic state in infected individuals, as the profile of genes positively associated with DWV-A resembles the profile upregulated in foragers but not nurses^42,43^. This was surprising, considering that the majority of bees in this meta-analysis were nurse-age, and thus considerably younger than the normal foraging age. Early-onset or “precocious” foraging, which represents an acceleration of behavioral maturation, has been linked to CCD via both computational and empirical studies^61,62^. We found this trend in workers but not drones, which do not forage. This is consistent with the finding that although high DWV titers have been identified in the drone endophallus, Yañez *et al*. demonstrated that infection does not impact drone mating flight performance^63^.

DWV-A infection was also associated with changes in the brain transcriptional regulatory architecture underlying behavioral maturation. Three transcription factors, *atf3*, *hairy*, and *cwo*, which our TRN model predicted to regulate many genes in the context of DWV-A infection, have previously also been computationally predicted to cause precocious foraging based on several different gene regulatory analyses of honey bee brains. For example, *atf3*, which is necessary for protein kinase A signaling^64^, was predicted to be involved in the initiation or maintenance of the foraging state^42^. Similarly, motifs associated with both *hairy* and *cwo* were found to be enriched in the promoter region of over half the genes upregulated in the brains of foragers relative to nurses and may therefore also be related to precocious foraging^42^. Our findings thus provide a suggestive link between viral infection and behavioral maturation, the latter of which has been linked to colony decline^36,61^. These three TFs in particular were implicated in Studies 2 and 4, which contain worker bees that were considerably younger than normal foraging age. Therefore, the impact of DWV-A on behavioral maturation may be exaggerated in younger bees who would not forage for several weeks under normal conditions. Perhaps DWV-A impacts transcriptional regulation differently in infected foragers, such as those in Study 1.

We suggest that these specific viral-associated neurogenomic effects on workers may promote horizontal viral transmission by facilitating spread between colonies. Foragers are more likely than nurses to accidentally enter (“drift”) into a foreign colony, and precocious foragers may be even more likely to drift than normal-age foragers. Precocious foraging also is associated with poor spatial memory relative to normal-age foragers^35^, and specific DWV-A induced learning deficits have been reported^26^. Moreover, the drifting of DWV-A infected workers may be more likely to occur under crowded modern apiary conditions. One interpretation of our findings is that DWV is manipulating host behavior to promote its spread. However, a variety of proximate stress factors cause precocious foraging, such as starvation^65^, infection by the protozoan *Nosema*^66^, *Varroa* mite infestation during development^67^, and a loss of normal-age foragers^34^, so it will be important to conduct additional studies to test this hypothesis. Given that precocious behavioral maturation is associated with colony decline^61,62^, understanding the evolutionary dynamics of the DWV-honey bee pathogen-host relationship is of considerable importance.

It was surprising to observe that scRNA-Seq analysis detected a cluster of cells in the brain with gene expression profiles that identify them as glia. We base this tentative conclusion on the observation that this cell cluster lacked the neuronal marker *elav* but was distinguished from other clusters by a relative abundance of *repo* and *Gs2*, two canonical markers of glia in insects^46,47^. In addition to expressing compelling molecular markers indicating glia, rather than neurons, we note that the small size of the cluster represents only ~3% of the ~2,200 sequenced cells, and glia in the insect brain have been estimated to number less than 10%, unlike the ~1:1 ratio split in vertebrates^68–70^. Because the scRNA-Seq samples were not infected with DWV-A, one possibility is that the molecular signature of DWV-A, common to all four datasets, is driven by gene expression primarily in activated glia cells. An alternative, nonexclusive, explanation is that infection stimulates molecular mechanisms in neurons that resemble the resting state of glia. In ladybird beetles (*Coleomegilla maculata*), infection with *Dinocampus coccinellae* paralysis virus (DcPV), which, like DWV, is also a picorna-like virus, similarly localized to glial cytoplasm in the brain, thus compromising host survival^71^. In the developing human brain, radial glia and astrocytes are more susceptible than neurons to infection by the flavivrus Zika^72^. Interestingly, we found *ebony*, which is activated in glia to generate circadian locomotion in *Drosophila*, to be positively associated with DWV-A in Studies 2 and 3 (Supplementary Tables S2 and S3, respectively). Circadian locomotion is a hallmark of honey bee foragers compared to nurses, which are arrhythmic^73^; thus, we speculate that glia may be playing a role in accelerating behavioral maturation. Because our results are derived from bioinformatic analyses of scRNA-Seq, we do not yet know the exact role of honey bee glia in the context of DWV infection. Further studies will be required to strengthen the link between glia-mediated viral response and its potential impact on behavior.

Given the accumulating evidence that picorna-like viruses cause cognitive impairment, in both vertebrates and invertebrates^26,74^, and given the strong effects of DWV-A on brain gene expression reported here, why was the DWV-A infection in all four studies covert, with no obvious effects on behavior? One possibility is that the behaviors of interest in those studies (reward responsiveness [Studies 1 and 3], aggression [Study 2], and caregiving [Study 4]) are subserved by different neural and molecular processes than those impacted by DWV-A infection. Another explanation is the advantage conferred to DWV-A via precocious foraging: infected bees that progress to a foraging state can do a better job spreading the virus between colonies than those that either succumb to infection or are ejected by their nestmates. Our findings thus far are specific to DWV-A, which is the predominant viral variant in North America^22,75^; future research will be necessary to determine if DWV-B and -C have similar impact on brain gene expression in adult honey bees. A better understanding of the ways in which DWV and other bee viruses affects brain and behavior will be important to future efforts aimed at reversing colony loss.

## Methods

### Datasets included in meta-analysis

We included 325 bees from four independent studies conducted at the University of Illinois Bee Research Facility in Urbana, IL between 2012 and 2014. For each study, source colonies containing honey bees were maintained according to standard beekeeping practices. Studies 1 and 3 used colonies headed by a naturally mated queen, and colonies for Studies 2 and 4 were headed by a queen instrumentally inseminated with semen from a single drone to reduce genetic variability between individuals. Study 3 only involved drones, which develop from unfertilized eggs and were therefore unaffected by queen insemination. Behavioral collections were made either directly from these colonies or from small cages of bees kept in a laboratory incubator that mimics in-hive conditions (34 ± 1 °C, 45 ± 10% relative humidity), as previously described^30,40,76^. No bees collected displayed physical symptoms of DWV (e.g., misshapen wings, immobility, differences in body size), nor did any show diminished responsiveness to investigators in the four respective studies. For collections, bees were flash-frozen in liquid nitrogen following a given experimental paradigm and stored at −80 °C until brain dissection and RNA extractions. Full experimental details, which apply to all four datasets, regarding sample collection, brain dissection, RNA extraction and RNA-Seq are described in the respective Methods sections of the two published datasets^29,40^, with the exception that Study 4 utilized the entire honey bee brain instead of the MB alone. All datasets are available for public access on the Gene Expression Omnibus database, respective accession IDs as follows: GSE130701 (Study 1), GSE85878 (Study 2), GSE130702 (Study 3), GSE130700 (Study 4).

### RNA-Sequencing analysis

We reprocessed original raw data for each dataset. Raw reads were mapped to the most recent *A. mellifera* genome assembly, build HAv3.1^77^, and the Deformed wing virus reference genome (GenBank accession number AJ489744^19^) using the STAR (v2.5.3) aligner with default settings^78^. Numbers of reads were counted using the featureCounts^79^ command in the Subread package (v1.5.2)^80^. Because we could not arbitrarily assign “high” and “low” levels of infection to datasets that are ubiquitously contaminated, we did not discretize samples by number of DWV reads. To normalize brain gene expression across the datasets, we calculated gene expression levels using %DWV (Fig. 1) as a covariate in a generalized linear model that included honey bee source colony as a blocking factor (for all experiments except Study 3, which only included a single colony) using edgeR^81^ (v3.24.3) in R (v3.5.2). We used the Benjamini-Hochberg (BH) correction for multiple testing^82^ and filtered lists of DWV-associated genes based on a threshold of FDR < 0.05.

### Gene list analyses

Gene list overlap analyses were conducted with the GeneOverlap^83^ and SuperExactTest^84^ packages in R. We used hypergeometric tests to calculate the statistical significance of observed gene list overlap compared to overlap expected by random chance, and calculated BH-corrected *p* values as noted in the text. A gene list universe of 8567 genes was used based on the 4-way overlap across the datasets. For Gene Ontology (GO) enrichment analysis, we first converted honey bee genes to *Drosophila melanogaster* orthologs via a one-to-one reciprocal best hit BLAST (v2.8.0; Supplementary Table 21)^85^. Lists containing genes differentially expressed in ≥ 2 datasets were then submitted to the GOrilla database and visualized using REVIGO^37,86,87^.

Lists of genes positively associated with DWV were then compared to published transcriptome data associated with nurse or foraging state. To obtain an unbiased list of nurse- and forager-upregulated genes, we used directionally concordant genes validated between two independent studies comparing nurses and foragers^42,43^. Pairwise comparisons were performed as described above.

### Transcriptional regulatory network construction

We used the Analyzing Subsets of Transcriptional Regulators Influencing eXpression (ASTRIX)^88^ approach to predict transcription factors (TFs) associated with DWV infection with an approach that used information from all four data sets. Using ASTRIX, we inferred a TRN by combining gene expression data from each study. A regulatory interaction was inferred via gene expression between a TF and its target based on co-expression: the predicted targets of TFs were defined as genes that share high mutual information (*p* < 10^−6^) with a TF and have a high predictive ability (correlation *r* > 0.8). A previous study^88^ validated ASTRIX-generated TF-target associations using data from ModENCODE^89^, REDfly^90^, and DroID^91^ databases. To assess the regulatory influence of each highly connected TF in the context of DWV infection, we tested for enrichment of its targets in each DWV-associated gene list using a Bonferroni FDR-corrected hypergeometric test with a cutoff of FDR < 0.05.

### ABC and CBA assay

We performed quantitative reverse transcription PCR (RT-qPCR) on 35 whole-brain samples from Study 4 (Fig. 3). RNA was extracted from whole brains using the RNeasy Mini Kit (Qiagen, Hilden, Germany) following overnight incubation in RNAlater-ICE (Invitrogen, Carlsbad, CA), as previously described^76^. RT-qPCR was performed on the RNA alongside a ten-fold dilution series of DWV variant-specific capsid or RdRp RNA standard curves using a Power SYBR® Green RNA-to-CT™ 1-Step Kit (Applied Biosystems, Waltham, MA). Each reaction contained 5 μL Power SYBR® Green RNA-to-CT PCR mix, 1 μL forward primer, 1 μL reverse primer for DWV-A, B or C Capsid [Highfield *et al*., *in preparation*] or RdRp region^45^, 0.08 μL reverse transcriptase, 1.92 μL RNase free H_2_O and 1 μL template RNA. The reverse transcription step occurred at 45 °C for 10 min and denaturation at 95 °C for 10 min, followed by 35 cycles of denaturizing at 95 °C for 15 sec, annealing at 58.5 °C for the capsid DWV-A, B, and C primers, RdRp DWV-A or B and at 61 °C for DWV-C RdRp primer for 15 sec and extension at 72 °C for 15 sec. A melt curve analysis was performed between 72 °C and 95 °C, at 0.1 °C increments, each with a 5 sec hold period. This step ensured that no contamination was present in the negative template controls and that only one product was amplified per primer set. Each sample was analyzed in duplicate. Copy numbers were determined using the following equation: *(Concentration RNA (ng/µL) × 6.022* × *10*^23^*) (Fragment length base pairs* × *109* × *325)*. Genome equivalents per bee were calculated using the following equation: *(Average copy number × RNA volume) x proportion of bee tissue* (see Figshare link for raw data).

### Single-cell RNA-Sequencing

We collected two seven-day-old adult worker honey bees from one source colony headed by a queen instrumentally inseminated with semen from a single drone (to increase genetic similarity among the bees). Brains were fresh-dissected in ice-cold Dulbecco’s phosphate-buffered saline (DPBS), and the mushroom boides (MB) were dissected in one sample. Both samples were then cut into small pieces and incubated for 2 min in an Eppendorf tube containing 90 μl of DPBS and 10 μl of 0.25% Trypsin with 2.21 mM EDTA. Next, 90 μl of the solution was removed and replaced with DPBS, and brain tissue was gently triturated with a pipette tip to dissociated cells. Fixation and rehydration were carried out in accordance with 10x protocols, with a 15 min methanol fixation on ice and storage at −20 °C for less than one week before rehydration and sequencing. scRNA-Seq libraries were constructed with the Chromium Single Cell 3′ Library and Gel Bead Kit v2 and were sequenced on a 2 × 150 nt lane on a HiSeq 4000 (Illumina, San Diego, CA). Raw and processed scRNA-Seq reads can be found under GEO accession ID GSE130785.

Raw scRNA-Seq reads were aligned to the honey bee genome (HAv3.1^77^) using Cell Ranger 3.0.1, and over 93% of sequenced reads mapped to the genome. For both WB and MB samples, we identified ~1,300 cells with an average of ~143,000 reads and a median of ~1,400 genes per cell. All following steps were performed with Seurat 3.0. We performed data normalization with default settings and combined the whole brain (WB) and MB datasets using the “merge” function, which predicted 13 cellular subpopulations via t-Distributed Stochastic Neighbor Embedding (tSNE) clustering. To identify the molecular profile of each cell population, we used the “FindAllMarkers” function in Seurat which performs a Wilcoxon Rank Sum Test to test for differentially expressed genes per tSNE cluster. We found no evidence of DWV contamination in either of our samples.

Next, we performed a series of hypergeometric tests to compare genes positively associated with DWV-A in the four analyzed datasets and genes upregulated in each of the 13 scRNA-Seq clusters using the SuperExactTest^83^ package. After performing BH correction for multiple comparisons, we reported significant FE at FDR < 0.05.

## Supplementary information

Dataset 1.

## Data Availability

Whole-tissue and scRNA-Seq data can be found on GEO, accession IDs presented in Table 1 and in Methods. R scripts for performing TRN analysis can be found at https://github.com/bukhariabbas/DWVTrn. All remaining raw data and R scripts can be accessed at 10.6084/m9.figshare.8341187.
